# The association of adverse reactions and depression in cervical cancer patients treated with radiotherapy and/or chemotherapy: moderated mediation models

**DOI:** 10.3389/fpsyg.2023.1207265

**Published:** 2023-08-08

**Authors:** Xiaoping Ding, Yingying Zhang, Jiaqing Wang, Ai Huang, Yilan Liu, Yanhong Han, Deying Hu

**Affiliations:** ^1^Department of Nursing, Union Hospital, Tongji Medical College, Huazhong University of Science and Technology, Wuhan, China; ^2^School of Nursing, Tongji Medical College, Huazhong University of Science and Technology, Wuhan, China; ^3^Department of Gynecologic Oncology, Union Hospital, Tongji Medical College, Huazhong University of Science and Technology, Wuhan, China

**Keywords:** depression, adverse reactions, cervical cancer, patient, moderation, mediation

## Abstract

**Objective:**

Several studies reported that adverse reactions to treatment, neuroticism, marital relations, and quality of life may impact the development of depression in cervical cancer patients treated with radiotherapy and/or chemotherapy, but the associations between them remained unclear. This study investigated the associations between these factors using moderated mediation models.

**Methods:**

Data were extracted from a survey involving cervical cancer patients treated with radiotherapy and/or chemotherapy at five tertiary hospitals in Hubei Province, China, from June to December 2022. The SPSS-PROCESS program was used to develop a moderated mediation model to study the roles of neuroticism, quality of life, and marital relations in the association between adverse reactions and depression in the study population.

**Results:**

A total of 802 cervical cancer patients treated with radiotherapy and/or chemotherapy (54.84 ± 9.68 years) were recruited. The prevalence of depression among these patients was 72.72%, with four symptom clusters of dizziness-ringing in the ears, digestive system-related symptoms, skin dryness and itching, and urinary frequency-urgency-leakage. Adverse reactions directly and positively affected the occurrence of depression, neuroticism mediated the association between adverse reactions and depression, while this association varied according to the quality of life and marital relations.

**Conclusion:**

Our findings suggest that depression is common among cervical cancer patients receiving radiotherapy and/or chemotherapy. Intervention targets for depression in cervical cancer patients should be precisely selected and targeted according to the quality of life and marital relations differences in patients, taking into account the cost of the intervention and the benefit to the patient.

## 1. Introduction

Depression is very common among cervical cancer patients, with multiple studies reporting rates of depressive symptoms in these patients ranging from 31.47 to 70.5% (Wang, 2014; Yang et al., 2017; Liu et al., 2021; Li, 2022). Additional research has shown that depression can prevent patients from adhering to treatments and adopting a healthy lifestyle, which can accelerate cancer progression, shorten survival, and lead to early death (Chida et al., 2008; Satin et al., 2009; Pinquart and Duberstein, 2010; Schneider and Moyer, 2010). After correcting for potential confounding factors including cancer site, disease stage, and socioeconomic status, Pinquart and Duberstein (2010) found that cancer patients with depression have a 22% increased risk of death compared to cancer patients without depression. Therefore, prevention and treatment of depression is essential to reducing mortality among cervical cancer patients, and achieve the WHO Global Strategy for the Elimination of Cervical Cancer (World Health Organization [WHO], 2020).

Adverse reactions of radiotherapy or chemotherapy may have a direct impact on the development of depression in patients with cervical cancer. Yu et al. (2018) reported that cervical cancer patients are more likely to develop negative emotional conditions, such as depression, after chemotherapy due to the adverse effects of treatment. Wang et al. (2017) and Malmsten et al. (2019) found that multiple adverse effects of radiotherapy, such as radiation vaginitis and radiation cystitis, pose a serious threat to the physical and mental health of cervical cancer patients. The Big Five personality theory categorizes personality traits into five major areas: extraversion, neuroticism, openness, agreeableness, and dutifulness, of which neuroticism is the personality trait that is closely related to depression. Several studies have established that the basic traits of neuroticism may be modified by intervention or cancer (Bleidorn et al., 2019; Roberts and Yoon, 2022), and levels of neuroticism are also associated with factors such as aging, increased physical illness, associated functional limitations, and reduced social resources (Watson and Clark, 1984; Hooker et al., 1998; Duberstein et al., 2003). The diagnosis of cervical cancer itself and the adverse reactions can result in decreased physiological function, increased vulnerability to the disease, and limited physical function, factors that may increase the level of neuroticism in patients with cervical cancer (Costa and McCrae, 1994). In addition, personality traits determine how individuals interpret and respond to potential stressors, thus helping to amplify or reduce the ultimate impact of the stressor (Zhang et al., 2018). Adverse reactions can be considered as stressor events themselves that threaten well-being and health. Whereas neuroticism is characterized by low self-esteem, shyness, anxiety, guilt, nervousness, moodiness, irrationality, and moodiness, highly neurotic individuals may have more negative emotions under exposure to stressors, and reduced resilience, which may lead to depressive symptoms (Malouff et al., 2005; Gong et al., 2020). Therefore, this study proposes hypothesis 1: adverse reactions of radiotherapy or chemotherapy may affect depression through the mediating role of neuroticism. The hypothesized conceptual model is shown in Supplementary Figure 1.

Quality of life refers to the perceived well-being of different domains of life, including physical, emotional, and social interactions related to illness or treatment, each of which is closely related to depression (U.S. Department of Health and Human Services FDA Center for Drug Evaluation and Research, U.S. Department of Health and Human Services FDA Center for Biologics Evaluation and Research, and U.S. Department of Health and Human Services FDA Center for Devices and Radiological Health, 2006; Haraldstad et al., 2019). Some studies have confirmed that the adverse reactions may have a direct impact on the development of depression in patients with cervical cancer, and that the association between adverse reactions and depression may be affected and enhanced when patients perceive that their quality of life is severely impaired during treatment and that their well-being will be greatly reduced (Meltzer-Brody and Davidson, 2000; Frick et al., 2007; Cotrena et al., 2016). Some studies have found that adverse reactions to cervical cancer treatment increase the level of neuroticism in patients with cervical cancer. If patients perceive a poorer quality of life due to a decline in somatic function and severe impacts on aspects of their physical health, they may have an impact on increasing their vulnerability to the disease, thus enhancing the impact of adverse treatment reactions on neuroticism levels in patients with cervical cancer. Highly neurotic patients with low self-esteem, shyness, anxiety, etc. may have more negative emotions under stressors, and if patients perceive enhanced emotional and social interactions, support and care from family and friends, etc., it may reduce the negative emotions and decrease the probability of depressive symptoms (Herzer et al., 2011; Ahn et al., 2016). Therefore, this study proposes hypothesis 2: the relationship among adverse reactions, neuroticism and depression is moderated by quality of life. The hypothesized conceptual mediation model is shown in Supplementary Figure 2.

Beach’s theory of marital discord depression suggests that critical support between couples includes physical and psychological support in the face of external stress, couple cohesion and joint activities, and acceptance of each other’s expression of feelings. Marital discord can lead to negative ways of interaction in the couple’s relationship, such as blame, severe put-downs, denigration, somatic and verbal aggression, and threats of separation and divorce, which contribute to the development and onset of depression (Hollist et al., 2007). Adverse reactions may have a direct impact on the development of depression in patients with cervical cancer. Studies found that 59.5% of cervical cancer patients reported no sexual life after treatment, and 78.0–98.5% of patients had adverse reactions such as difficulty in sexual intercourse, vaginal dryness, and decreased sexual interest, which brought great psychological pressure to the couple life of cervical cancer patients (Corrêa et al., 2016; Zhou et al., 2016; Zhen et al., 2019). However, a good marital relationship can provide good physical and psychological support and maintain patients’ self-esteem. Then the direct effect of adverse reactions on depression would be diminished (Kronmüller et al., 2011). Similarly, the effect of adverse reactions on neuroticism can be diminished if the spouse accompanies the patient and jointly participates in the cancer treatment process so that the patient feels the reliability, availability, and stability of the spouse (Heene et al., 2007). Conversely, since highly neurotic patients with characteristics such as low self-esteem, shyness, and anxiety may experience more negative emotions under exposure to stressors, if the spouse then displays negative interactions such as blame, severe put-downs, denigration, somatic and verbal aggression, and threats of separation and divorce, it may further exacerbate the patient’s negative emotions and increase the probability of depressive symptoms (Ahn et al., 2016). Therefore, this study proposes hypothesis 3: the relationship among adverse reactions, neuroticism, and depression is moderated by marital relations. The hypothesized conceptual mediation model is shown in Supplementary Figure 3.

These above factors have been shown to impact the development of depression in cervical cancer patients treated with radiotherapy and/or chemotherapy, but the underlying associations remain unclear. Therefore, the present study constructed a moderated mediation model based on hypotheses 1–3, whose hypothesized model is shown in Supplementary Figure 4, to test the following three hypotheses: (1) adverse reactions, neuroticism, marital relations, and quality of life have an impact on the occurrence of depression in cervical cancer patients; (2) neuroticism mediates the relationship between adverse reactions and depression; and (3) the relationship among adverse reactions, neuroticism, and depression is moderated by both quality of life and marital relations. This investigation of the association by which adverse reactions influence depression in cervical cancer patients treated with radiotherapy and/or chemotherapy in a developing country provides theoretical and empirical support for methods to prevent and treat depression in cervical cancer patients in order to reduce mortality among cervical cancer patients.

## 2. Materials and methods

### 2.1. Study design and participants

This cross-sectional study was conducted from June to December 2022. A previous study reported that Hubei Province may be an area with a high prevalence of depression among cancer patients in mainland China (Ding et al., 2023). In view of the feasibility of the study, participants in this study were selected from four tertiary general hospitals and one tertiary specialist oncology hospital in Hubei Province in central China. These five hospitals are important medical institutions for treating gynecological oncology patients in Hubei Province and to some extent represent the overall situation in Hubei Province.

Convenience sampling was used to recruit participants who met the following criteria: (1) pathologically confirmed primary cervical cancer; (2) age ≥18 years; (3) previously received or currently receiving radiotherapy and/or chemotherapy as a treatment modality; (4) stable vital signs; (5) ability to read and understand Chinese questionnaires as well as communicate verbally; and (6) willingness to voluntarily participate in the study and sign the informed consent form. Patients were excluded if they had severe cognitive impairment, critical illness, or other types of cancer as comorbidities, or if they were unaware of their cancer diagnosis.

Based on the design for the current study and the requirement that outcome indicators be categorical variables, and considering that previous studies have reported that 53.91% of gynecological cancer patients in mainland China have depressive symptoms (Ding et al., 2023), with an allowable absolute error of 5 and 95% confidence limits and assuming a 10–20% lost-to-review rate, we calculated that at least 503 questionnaires needed to be distributed.

This study protocol was approved by the Clinical Ethics Committee of Huazhong University of Science and Technology approval (2022S060), and patients with cervical cancer who had elevated Patient Health Questionnaire 9 (PHQ-9) scores and suicidal thoughts were encouraged and assisted to seek mental health care.

### 2.2. Measures

#### 2.2.1. General information questionnaire for cervical cancer patients

This questionnaire collected data regarding the hospital visited, patient age, education level, marital status, occupational status, stage of cervical cancer, number of chemotherapy sessions completed, and number of radiotherapy sessions completed.

#### 2.2.2. Patient-Reported Outcomes version of the Common Terminology Criteria for Adverse Events

This scale provides a database of 124 entries measuring 78 symptomatic adverse events, which can be freely combined by the investigator for assessment according to the type of cancer. Using 1–3 questions, each adverse event is assessed in terms of “frequency,” “severity,” and “interference with daily activities,” and a composite score is calculated for each adverse event based on a map of 179 combinations of scores, ranging from 0 to 3 for each question (National Cancer Institute [NIH], 2022). Through literature research and discussions with gynecological oncologists, we identified 21 adverse events most commonly experienced in patients with cervical cancer treated with radiotherapy, including vomiting, nausea, decreased appetite, fatigue, constipation, dry mouth, edema, chills, dizziness, ringing in ears, hair loss, numbness, pain, vaginal bleeding, vaginal dryness, skin dryness, itching, urinary frequency, urinary urgency, urinary leakage, and painful urination. The Cronbach’s α, convergent validity [average variance extracted, (AVE)] and variance contributions of the first factors of the Patient-Reported Outcomes version of the Common Terminology Criteria for Adverse Events (PRO-CTCAE) in this study were 0.897, 0.523, and 23.13%, respectively.

#### 2.2.3. Functional Assessment of Cancer Therapy-Cervix

This scale is used to assess patients’ quality of life and consists of the Functional Assessment of Cancer Therapy-General (FACT-G) and the Cervix subscale (Cx subscale). The FACT-G has 27 items with four dimensions, including: physical status (7 items), social/family status (7 items), emotional status (6 items), and functional status (7 items). The Cx subscale consists of 15 items. All items are scored on a 5-point Likert scale (range, 0–4), which correspond to responses of not at all, a little, somewhat, quite, and very much, respectively. The total score for all entries is summed and ranges from 0 to 168, with a higher score indicating better quality of life (Yu et al., 2000). The Cronbach’s α, convergent validity (AVE) and variance contributions of the first factors of the FACT-Cx in this study were 0.917, 0.584, and 24.52%, respectively.

#### 2.2.4. Patient Health Questionnaire 9

The Patient Health Questionnaire 9 (PHQ-9) is used to assess the severity of depression, with scores ranging from 0 to 3 for each item, which represent not at all, several days, more than half the days, and almost every day, respectively. The scores for all items are summed to give a total score, which ranges from 0 to 27. The final scores are ranked as: 0–4, no depression; 5–9, possible mild depression; 10–14, possible moderate depression; 15–19, possible moderate to severe depression; and 20–27, possible severe depression (Kroenke et al., 2001). The Cronbach’s α, convergent validity (AVE) and variance contributions of the first factors of the PHQ-9 in this study were 0.893, 0.521, and 30.08%, respectively.

#### 2.2.5. NEO-Five Factor Inventory-Neuroticism Subscale

This scale has 12 items that are scored on a 5-point scale, with scores of 1–5 indicating strongly disagree, largely disagree, no opinion, largely agree, and strongly agree, respectively. A higher score corresponds to more pronounced the neuroticism (Yang et al., 1999). The Cronbach’s α, convergent validity (AVE) and variance contributions of the first factors of the NEO-Five Factor Inventory-Neuroticism Subscale (NEO-FFIN) in this study were 0.841, 0.518, and 28.98%, respectively.

#### 2.2.6. Locke-Wallace Short Marital Adjustment Test

The Locke-Wallace Short Marital Adjustment Test (LWSMAT) is used to assess patients’ level of perceived marital relationship. The scale consists of 15 items with a total score range of 2–158. A higher score indicates better quality of the marital relations (Wang et al., 1999). The Cronbach’s α, convergent validity (AVE) and variance contributions of the first factors of the LWSMAT in this study were 0.825, 0.511, and 25.46%, respectively.

### 2.3. Data collection

Data collection was undertaken by the researcher herself, who is qualified as a senior counselor, and three gynecological oncology nurses, all of whom have more than 5 years of experience working with gynecological cancer patients. Prior to the study, all participants were informed of the purpose and procedures of the study. The survey process fully respected the patient’s right to informed, voluntary, and unconditional withdrawal. Upon completion of the questionnaires, participants received RMB20 as compensation. Of 812 questionnaires eventually distributed, 809 were collected. Exclusion of 7 unqualified questionnaires left a total of 802 valid questionnaires for analysis. All data were entered in duplicate copies and passed the consistency test to ensure the accuracy of data entry.

### 2.4. Data analysis

SPSS 26.0 software was used for descriptive statistical analysis, Pearson’s correlations analysis was used to measure the relationship between five continuous variables (neuroticism, marital relations, quality of life, adverse reactions, and depression). Secondly, the qgraph package in R software (4.1.0) was used to construct a symptom network graph based on the EBICglasso function, in which the symptoms are nodes and the lines connecting the nodes are the edges of the network, with thicker edges representing stronger correlation between the two symptoms. Finally, the SPSS-PROCESS (Model 76) program (Hayes, 2013) was used to test the mediation model of multilateral regulation to study the roles of neuroticism, marital relations, and quality of life in the association between adverse reactions and depression among cervical cancer patients treated with radiotherapy and/or chemotherapy.

## 3. Results

### 3.1. Descriptive statistics and correlations among variables

Overall, 72.72% of the patients with cervical cancer reported depressive symptoms, of which 359 (44.76%) reported mild depression, 144 (17.96%) moderate depression, and 80 (10%) moderately severe or greater depression. The mean age of participants was 54.84 ± 9.68 years (range, 23–84 years). The mean number of chemotherapy sessions completed was 4.57 ± 9.68, and the mean number of radiotherapy sessions completed was 16.75 ± 14.41. More than half of the study participants had a junior high school education or less (63.0%, *n* = 505), were married (84.2%, *n* = 675), and were unemployed (55.4%, *n* = 444). With respect to cancer staging, 53.2% of participants were diagnosed with stage II cervical cancer (*n* = 427) and 29.2% had stage III cervical cancer (*n* = 234).

Pearson correlation analysis of the variables showed that depression in cervical cancer patients was significantly positively correlated with adverse reactions and neuroticism (*p* < 0.01) and negatively correlated with marital relations and quality of life (*p* < 0.01; Supplementary Table 1).

### 3.2. Identification of core symptom clusters in cervical cancer patients treated with radiotherapy and/or chemotherapy

**FIGURE 1:**
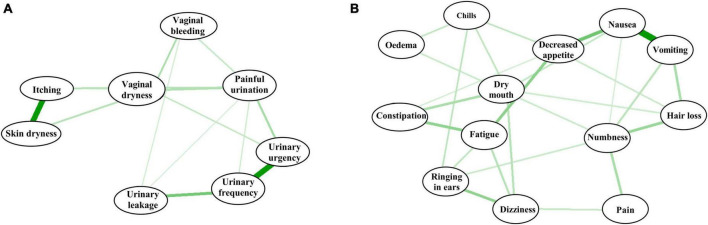
Networks of symptoms experienced by cervical cancer patients treated with radiotherapy **(A)** and chemotherapy **(B)**.

A total of 82.4% of cervical cancer patients in this study had stage II–III disease, and the use of multiple treatment modalities is known to lead to multiple adverse reactions occurring simultaneously and interacting with each other to form symptom clusters (Dodd et al., 2001; Rha and Lee, 2021), the specificity of the impact of core symptom clusters on depression also needs to be considered. Therefore, this study identified core symptom clusters based on the coarseness of the edges in the symptom network (Figure 1) and found that: among the adverse effects of radiotherapy, the symptoms with strong correlations were skin dryness and itching (*r* = 0.973) and urinary frequency-urgency-leakage (*r* = 0.813) (Figure 1A); and among the adverse effects of chemotherapy, the more relevant symptoms were vomiting-nausea-decreased appetite-fatigue-constipation-dry mouth (*r* = 0.746) and dizziness-ringing in the ears (*r* = 0.718) (Figure 1B). Based on the characteristics of these symptom clusters, vomiting-nausea-decreased appetite-fatigue-constipation-dry mouth was named the digestive system-related symptom cluster.

### 3.3. Associations by which adverse reactions, neuroticism, marital relations, and quality of life influence depression in cervical cancer patients treated with radiotherapy and/or chemotherapy

Cervical cancer stage, chemotherapy course, and number of radiation treatments were included as control variables in the regression analysis, and the total scores for the digestive system-related symptom cluster, dizziness-ringing in the ears, skin dryness and itching, and urinary frequency-urgency-leakage were included as independent variables. Additionally, PHQ-9 scale scores were treated as dependent variables, NEO-FFIN scale scores as mediating variables, and FACT-Cx and LWSMAT scores as moderating variables in constructing a mediated effects model with multilateral moderation. As presented in Table 1, the goodness of fit of Models 1–4 ranged from 0.548 to 0.637, with all models passing the *F*-test with a significance level of 0.05, which indicates a good fit and the ability to reflect the hypothetical mechanism of this study.

**TABLE 1:** Regression coefficients for moderated mediation models.

		Model 1	Model 2	Model 3	Model 4
Control variables	Cervical cancer stage	0.0191 (−0.2945 to ∼0.3328)	0.0576 (−0.2755 to ∼0.3906)	0.0842 (−0.2459 to ∼0.4144)	0.0519 (−0.2974 to ∼0.4012)
	Chemotherapy course	0.0371 (−0.0612 to ∼0.1354)	0.1058 (0.004∼0.2075)	0.1056* (0.0047∼0.2065)	0.1638* (0.0579∼0.2697)
	Number of radiation treatments	−0.017 (−0.0349 to ∼0.0009)	−0.0199 (−0.039 to ∼−0.0008)	−0.019 (−0.0382 to ∼0.0003)	−0.0276 (−0.0479 to ∼−0.0073)
Independent variables	Digestive system-related symptom cluster	0.0225* (0.0104∼0.1489)			
	Dizziness-ringing in the ears		0.347* (0.1964∼0.5023)		
	Skin dryness and itching			0.5715* (0.2385∼0.9045)	
	Urinary frequency-urgency-leakage				0.4003* (0.0999∼0.6992)
Mediating variables	Neuroticism	0.3321** (0.1527∼0.5115)	0.2417** (0.0589∼0.4246)	0.4146** (0.2343∼0.5949)	0.275* (0.0891∼0.4609)
Moderating variables	Quality of life	−0.0302 (−0.0841 to ∼0.0237)	−0.0759** (−0.1365 to ∼−0.0154)	−0.0194 (−0.0776 to ∼0.0388)	−0.05 (−0.111 to ∼0.0109)
	Marital relations	−0.0291 (−0.0792 to ∼0.0209)	−0.0345 (−0.0895 to ∼0.0204)	−0.0571* (−0.1103 to ∼−0.0039)	−0.0512 (−0.1075 to ∼0.005)
Interactions	Adverse reactions × marital relations	0.0009 (−0.0018 to ∼0.0035)	−0.0031 (−0.0117 to ∼0.0055)	0.0004 (−0.0051 to ∼0.006)	0.0013 (−0.0039 to ∼0.0064)
	Neuroticism × marital relations	0.0005 (−0.001 to ∼0.002)	0.001 (−0.0006 to ∼0.0026)	0.0015* (0.0001∼0.003)	0.0012 (−0.0004 to ∼0.0028)
	Adverse reactions × quality of life	0.0033 (−0.0001 to ∼0.0067)	0.0144** (0.0041∼0.0247)	0.0176** (0.0105∼0.0246)	0.0048 (−0.0018 to ∼0.0114)
	Neuroticism × quality of life	−0.0013 (−0.003 to ∼0.0004)	−0.0007 (−0.0026 to ∼0.0011)	−0.0032** (−0.0049 to ∼−0.0015)	−0.0015 (−0.0034 to ∼0.0003)
*R* ^2^		0.637	0.574	0.560	0.548
Significant		<0.001	<0.001	<0.001	<0.001
*F* value		102.400	81.517	80.801	71.660
Dependent variable: depression					

**p < 0.01, *p < 0.05.

In Model 1, regardless of the patient’s quality of life and marital relations, the indirect effect between the digestive system-related symptom cluster and depression was not significant, but the digestive system-related symptom cluster affected depression directly and positively. In Model 2, regardless of the patient’s quality of life and marital relations, the indirect effect between the dizziness-ringing in the ears and depression was not significant, but the dizziness-ringing in the ears affected depression directly and positively. These results meant that the relationship between the digestive system-related symptom cluster/the dizziness-ringing in the ears, neuroticism, and depression was not moderated by patient’s quality of life and marital relations. In Model 3, regardless of the patient’s quality of life and marital relations, the indirect effect between the skin dryness and itching and depression was not significant. However, among patients with low marital relations/high quality of life and high marital relations/high quality of life, the skin dryness and itching could directly and positively affect depression. These results meant that the relationship between the skin dryness and itching, neuroticism, and depression can be significantly moderated by quality of life and marital relations. In Model 4, the indirect effect between the urinary frequency-urgency-leakage and depression was significant, except for those patient with low marital relations/high quality of life. In addition, among patients with low marital relations/high quality of life and high marital relations/high quality of life, the urinary frequency-urgency-leakage could directly and positively affect depression. These results meant that the relationship between the urinary frequency-urgency-leakage, neuroticism, and depression can be significantly moderated by quality of life and marital relations.

**FIGURE 2:**
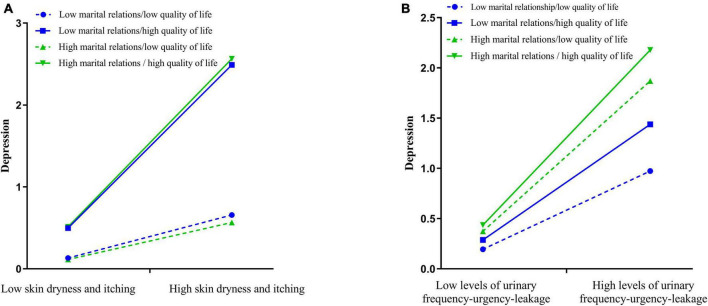
Visualization of moderated mediation effect in skin dryness and itching and depression **(A)**, and urinary frequency-urgency-leakage and depression **(B)**.

The total effect values of Models 3 and 4 in Table 2 were used as slopes and the scores of marital relations and quality of life were divided into high and low values by quartiles to plot the moderating effects of marital relations and quality of life (Figure 2) to visualize the results of Models 3 and 4 above.

**TABLE 2:** Indirect effects moderated by marital relations and quality of life.

		Marital relations			
	Effect type	Low	Low	High	High
		Quality of life			
		Low	High	Low	High
Model 1	Indirect	0.0453 (−0.003 to ∼0.105)	0.0024 (−0.049 to ∼0.063)	0.0566 (−0.007 to ∼0.132)	0.0093 (−0.043 to ∼0.062)
	Direct	0.3605** (0.267∼0.454)	0.473** (0.344∼0.602)	0.3947** (0.282∼0.508)	0.5072** (0.407∼0.608)
	Total	0.4058** (0.264∼0.563)	0.4754** (0.295∼0.665)	0.4513** (0.274∼0.640)	0.5165** (0.364∼0.670)
Model 2	Indirect	0.0009 (−0.010 to ∼0.103)	−0.077 (−0.242 to ∼0.075)	0.1014 (−0.058 to ∼0.270)	0.0031 (−0.161 to ∼0.176)
	Direct	0.5704** (0.332∼0.809)	1.053** (0.814∼1.452)	0.4564* (0.105∼0.808)	0.939** (0.816∼1.262)
	Total	0.5713** (0.232∼0.811)	0.976** (0.842∼1.528)	0.5578* (0.047∼0.878)	0.9421** (0.895∼1.438)
Model 3	Indirect	0.0297 (−0.112 to ∼0.058)	0.016 (−0.097 to ∼0.071)	0.0271 (−0.143 to ∼0.090)	0.0154 (−0.109 to ∼0.082)
	Direct	0.1017 (−0.287 to ∼0.083)	0.482** (0.183∼0.782)	0.086 (−0.295 to ∼0.123)	0.4976** (0.248∼0.747)
	Total	0.1314 (−0.399 to ∼0.142)	0.498** (0.186∼0.853)	0.1131 (−0.438 to ∼0.213)	0.513** (0.240∼0.829)
Model 4	Indirect	0.1049* (0.036∼0.183)	0.0351 (−0.048 to ∼0.129)	0.2371* (0.137∼0.358)	0.1359* (0.043∼0.245)
	Direct	0.0898 (−0.060 to ∼0.239)	0.2525** (0.001∼0.504)	0.137 (−0.057 to ∼0.331)	0.2997** (0.101∼0.499)
	Total	0.1947* (0.024∼0.423)	0.2876** (0.047∼0.433)	0.3741* (0.080∼0.689)	0.4356** (0.143∼0.743)

**p < 0.01, *p < 0.05. In Models 1–4, the Patient Health Questionnaire 9 (PHQ-9) scale score is a dependent variable; the NEO-Five Factor Inventory-Neuroticism Subscale (NEO-FFIN) score is a mediating variable; and the Functional Assessment of Cancer Therapy-Cervix (FACT-Cx) and Locke-Wallace Short Marital Adjustment Test (LWSMAT) scores are moderating variables. In Model 1, the digestive system-related symptom cluster is the independent variable; in Model 2, dizziness-ringing in the ears is the independent variable; in Model 3, skin dryness and itching is the independent variable; and in Model 4, urinary frequency-urgency-leakage is the independent variable.

### 3.4. Robustness analysis

#### 3.4.1. Using the moderator variable social support instead of marital relations

First, we found that the social support was closely connected with marital relations, for the reason that the marriage relationship is one of the most important social relationships (Cohen et al., 2000). Cervical cancer stage, chemotherapy course, and number of radiation treatments were included as control variables in the regression analysis, and the total scores for the digestive system-related symptom cluster, dizziness-ringing in the ears, skin dryness and itching, and urinary frequency-urgency-leakage were included as independent variables. Additionally, PHQ-9 scale scores were treated as dependent variables, NEO-FFIN scale scores as mediating variables, and FACT-Cx and Perceived Social Support Scale scores (Zimet et al., 1990) as moderating variables in constructing moderated mediation models. As presented in Supplementary Table 2a, the goodness of fit of Models 1–4 ranged from 0.569 to 0.611, with all models passing the *F*-test with a significance level of 0.05, which indicates the four models fitted well. More importantly, as presented in Supplementary Table 2b, the relationship between dry skin dryness and itching/urinary frequency-urgency-leakage, neuroticism, and depression was significantly moderated by social support and quality of life.

#### 3.4.2. Using maximum likelihood estimation instead of least squares estimation

All the results of this study were implemented based on the SPSS software process plug-in, which has a least squares estimation (OLS) method for parameter estimation (Hayes, 2013). We used the maximum likelihood estimation to verify the main results again, and the software chosen was AMOS 23.0. As presented in Supplementary Table 3a, the values of Chi-square/df in Models 1–4 ranged from 1.983 to 2.873, the values of CFI in Models 1–4 ranged from 0.900 to 0.912, the values of AGFI in Models 1–4 ranged from 0.902 to 0.916, the values of RMSEA in Models 1–4 ranged from 0.064 to 0.072, which indicates the four models fitted well. More importantly, as presented in Supplementary Table 3b, the relationship between dry skin dryness and itching/urinary frequency-urgency-leakage, neuroticism, and depression was significantly moderated by social support and quality of life.

## 4. Discussion

The mean age of the cervical cancer patients treated with radiotherapy and/or chemotherapy included in this study was 54.84 ± 9.68 years, and most of the patients were from rural areas, married, unemployed, or working in agriculture. The data show that the incidence of cervical cancer in Chinese women peaks in the 50–54 age group and declines thereafter (Zhang, 2021; Cai et al., 2023). The generally low level of literacy may be related to patients’ knowledge base, awareness of medical examinations, and disease perception. Studies have shown that the lower the literacy level of cancer patients, the lower their health literacy and the higher the health risks they face (Sentell and Braun, 2012).

Several new findings emerged from this study, first, the prevalence of depression among cervical cancer patients treated with radiotherapy and/or chemotherapy in this study was high and much higher than the national average for the prevalence of depression among all cancer patients in China of 44.63% (Ding et al., 2023). This high prevalence of depression may be due to the fact that the majority of cervical cancer cases in this study were at an advanced stage of disease, and treatment of advanced cervical cancer with radical radiotherapy combined with platinum-based concurrent chemotherapy can make patients suffer from multiple symptoms and thus be more likely to experience depression (Jiang et al., 2023). Our findings suggest that depression in cervical cancer patients needs to be addressed and taken into account in clinical practice.

Secondly, the results of Models 1 and 2 in Table 2 suggest that the digestive system-related symptom cluster and dizziness-ringing in the ears can directly and significantly positively affect depression in patients with cervical cancer treated with radiotherapy and/or chemotherapy. The co-occurrence of the digestive system-related symptom cluster may be related to the cytotoxicity of chemotherapeutic drugs, which stimulate the medullary vomiting center and transmit the corresponding signals through peripheral and central pathways, leading to nausea and vomiting. When patients feel nauseous, gastric tension, and peristalsis are reduced and duodenal tone is increased, which can be accompanied by gastric discomfort and cause loss of appetite. Frequent vomiting can also leave patients with a dry mouth. Antiemetics are often used in clinical practice to inhibit bowel movements to relieve these symptoms, which in turn may cause constipation, and thus, the synergistic effect of these clusters of adverse gastrointestinal symptoms creates a significant psychological burden for patients with cervical cancer (Saito and Tsukuda, 2010; Elshoff et al., 2015; Cherwin and Perkhounkova, 2017). Dizziness-ringing in the ears may be related to peripheral neurotoxicity involving the nerves in the brain caused by the platinum-based chemotherapy regimen in patients with cervical cancer, and studies have shown that the cluster of neurological symptoms can lead to psychological problems such as depression, forcing patients to stop chemotherapy prematurely, thereby reducing the effectiveness of anticancer treatment, and possibly reducing overall survival (Ezendam et al., 2014; Tanay et al., 2017). Therefore, it is recommended that, given the limited mental health resources in Chinese oncology medical institutions, interventions for depression should target interventions to alleviate symptoms if digestive system-related symptom cluster and dizziness-ringing in the ears are present in order to improve intervention efficiency and reduce healthcare expenditure.

Thirdly, in the results of Model 3 in Table 2, the relationship between the skin dryness and itching, neuroticism, and depression can be significantly moderated by marital relations and quality of life. More specifically, among patients with low marital relations/high quality of life and high marital relations/high quality of life, the skin dryness and itching could directly and positively affect depression. Skin dryness and itching in patients with cervical cancer is mainly caused by inflammatory skin reactions caused by radiation exposure, which not only leads to changes in physical appearance and lowers patients’ self-esteem levels, but also significantly reduces patients’ quality of life and causes significant psychological burden (Chu et al., 2021; Asada et al., 2022), with studies reporting insomnia in over 88% of patients with pruritic skin. Pruritic skin can significantly reduce patients’ quality of life, and with pruritic skin disease, patients are 1.4 times more likely to suffer from depressive mood or anxiety than those who do not (Kopyciok et al., 2016; Huet et al., 2022). Therefore, taking into account the cost of intervention and patient benefit, it is recommended that in clinical psycho-oncology practice, the target of depression interventions when skin dryness and itching are present should be determined based on the specific quality of life and marital relations status of the cervical cancer patient. That for patients with low marital relations/high quality of life and those with high marital relations/high quality of life, depression interventions should be aimed at alleviating skin dryness and itching is a key target to improve the efficiency of interventions and reduce healthcare expenditure.

Fourthly, due to its anatomical location next to each other, the bladder can be damaged during radiation control of the tumor leading to radiation cystitis, inflammatory reactions such as urinary frequency, urinary urgency and leakage, and in severe cases, vesicovaginal fistula or even death, posing a significant threat to the patient’s psychological health (Rehailia-Blanchard et al., 2019; Chen et al., 2021). The results of Model 4 in Table 2 suggest that the relationship between urinary frequency-urgency-leakage, neuroticism, and depression can be significantly moderated by marital relations and quality of life, but that this relationship varies by the quality of life and marital relations differences in patients. Specifically, the indirect effect between the urinary frequency-urgency-leakage and depression was significant, except for those patient with low marital relations/high quality of life. In addition, among patients with low marital relations/high quality of life and high marital relations/high quality of life, the urinary frequency-urgency-leakage could directly and positively affect depression. These results suggest that in clinical psycho-oncology practice, for patients with cervical cancer who have urinary frequency-urgency-leakage, interventions for depression may be focused differently depending on the quality of life and marital relations differences in patients, taking into account the cost and benefit of the intervention. In patients with low marital relations/high quality of life, depression interventions should be targeted at alleviating urinary frequency-urgency-leakage; in patients with high marital relations/high quality of life, depression interventions should focus on both neuroticism and urinary frequency-urgency-leakage.

## 5. Conclusion

Depression is common among cervical cancer patients treated with radiotherapy and/or chemotherapy, with four core symptom clusters of dizziness-ringing in the ears, digestive system-related symptoms, skin dryness and itching, and urinary frequency-urgency-leakage. The relationship between the four symptom clusters, neuroticism and depression is variable, and this relationship changes depending on the quality of life and marital relations. In the context of limited mental health resources in Chinese oncology medical institutions, the intervention targets for depression in cervical cancer patients should be precisely selected and targeted according to the quality of life and marital relations differences in the population, taking into account the cost of the intervention and the benefit to the patient. This will improve the efficiency of interventions and reduce healthcare expenditure.

## 6. Limitation

Firstly, the patients were all selected from the same province in China, but significant differences in depression among cancer patients have been reported among different provinces (Ding et al., 2023). Therefore, future research is needed to test the generalizability of the findings of this study. Secondly, cross-sectional studies cannot confirm causality, and future longitudinal study designs could be used to further deduce the associations of action between influencing variables and depression in cervical cancer patients. Thirdly, the occurrence of depression in cervical cancer patients may also be related to genetic or biological factors, and more detailed studies incorporating such factors could be conducted in the future.

## Data availability statement

The raw data supporting the conclusions of this article will be made available by the authors, without undue reservation.

## Ethics statement

The studies involving human participants were reviewed and approved by the Clinical Ethics Committee of Huazhong University of Science and Technology approval (2022S060). The patients/participants provided their written informed consent to participate in this study.

## Author contributions

DH and XD: conceptualization. XD, DH, and YZ: methodology and writing—review and editing. XD, YZ, and JW: software, validation, and investigation. XD and YZ: formal analysis. DH, YL, AH, and YH: resources and supervision. XD: data curation and writing—original draft preparation. DH, YL, and AH: project administration. All authors had read and agreed to the published version of the manuscript.
