# Supplementary material for: The association of adverse reactions and depression in cervical cancer patients treated with radiotherapy and/or chemotherapy: moderated mediation models
Source: Front Psychol. 2023 Aug 8;14:1207265. doi: 10.3389/fpsyg.2023.1207265 (PMC10442555; doi:10.3389/fpsyg.2023.1207265)
Supplement: Supplementary file 1 [file Data_Sheet_1.docx]

**Supplementary Materials**

Supplementary Figure 1: A hypothesized conceptual model of mediator variable neuroticism causally located between adverse reactions and depression


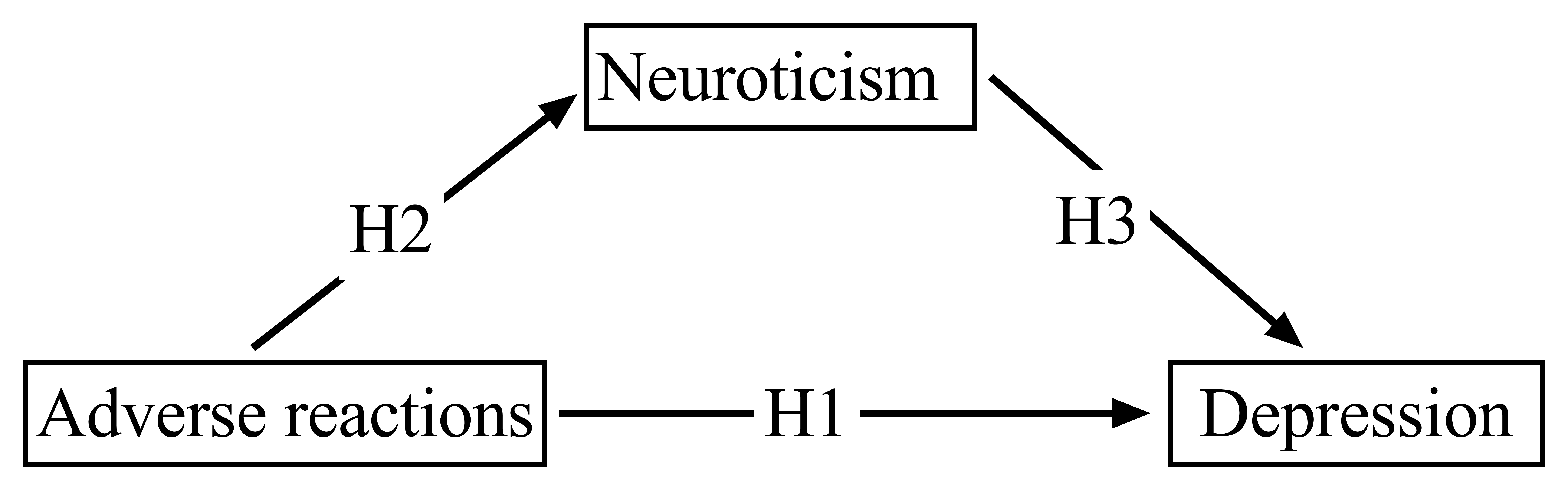


Hypothesis H1: Adverse reactions of radiotherapy or chemotherapy can have an impact on depression in patients with cervical cancer；

Hypothesis H2: Adverse reactions of radiotherapy or chemotherapy can have an impact on neuroticism in patients with cervical cancer；

Hypothesis H3: Neuroticism can have an impact on depression in patients with cervical cancer.

Supplementary Figure 2: A hypothesized conceptual mediation model with all three paths moderated by a common moderator variable quality of life


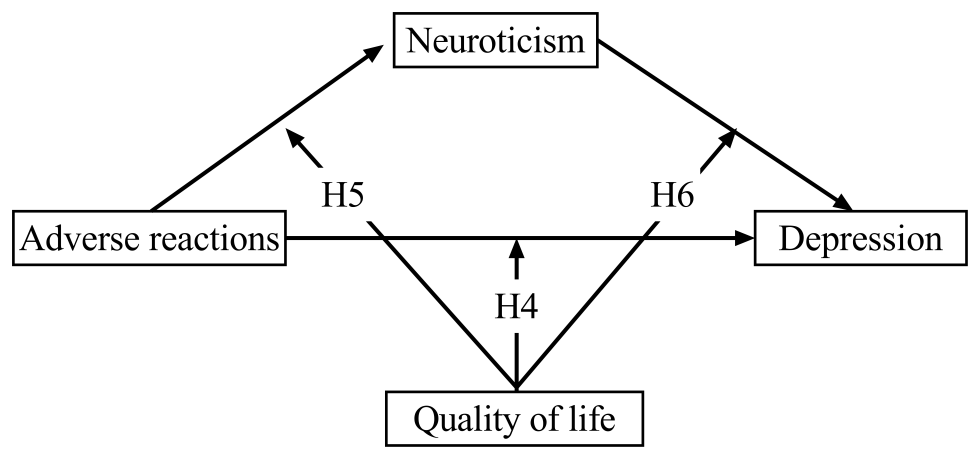


Hypothesis H4: The relationship between adverse reactions and depression can be moderated by quality of life；

Hypothesis H5: The relationship between adverse reactions and neuroticism can be moderated by quality of life;

Hypothesis H6: The relationship between neuroticism and depression can be moderated by quality of life.

Supplementary Figure 3: A hypothesized conceptual mediation model with all three paths moderated by a common moderator variable marital relations


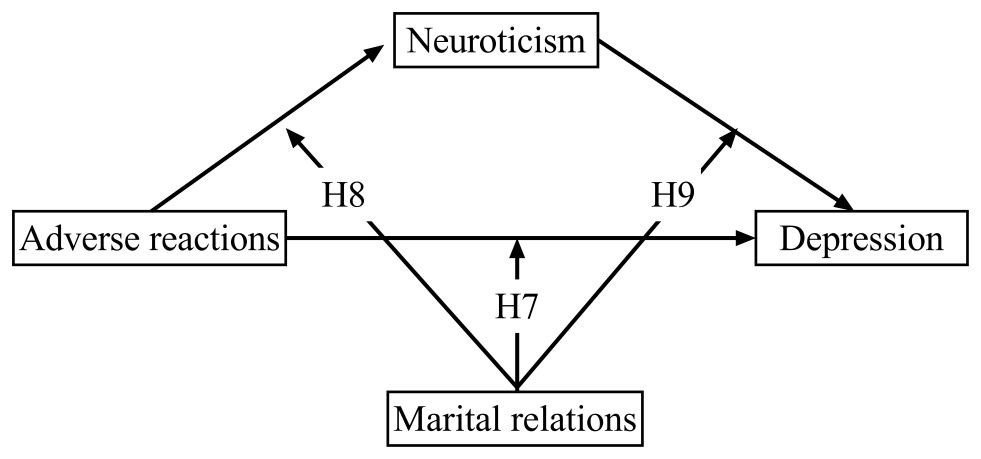


Hypothesis H7: The relationship between adverse reactions and depression can be moderated by marital relations；

Hypothesis H8: The relationship between adverse reactions and neuroticism can be moderated by marital relations；

Hypothesis H9: The relationship between neuroticism and depression can be moderated by marital relations.

Supplementary Figure 4: A hypothesized conceptual moderated mediation model of the association between adverse reactions and depression in patients with cervical cancer treated with radiotherapy and/or chemotherapy

**
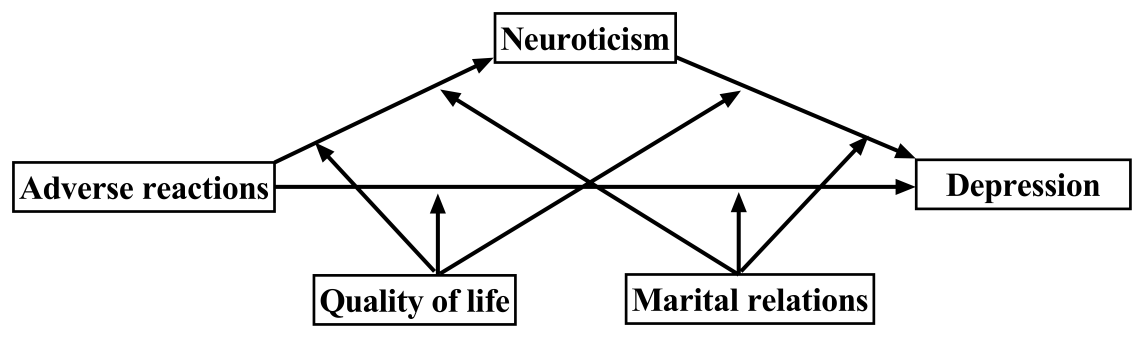
**

Supplementary Table 1: Descriptive statistics and correlation analysis between variables (N=802).

| Variables | M | SD | Adverse reactions | Neuroticism | Marital relations | Quality of life | Depression |
| --- | --- | --- | --- | --- | --- | --- | --- |
| Adverse reactions | 17.41 | 11.26 | 1 | 0.581** | -0.316** | -0.713** | 0.682** |
| Neuroticism | 32.97 | 7.80 | 0.581** | 1 | -0.418** | -0.657** | 0.673** |
| Marital relations | 100.81 | 24.16 | -0.316** | -0.418** | 1 | 0.399** | -0.352** |
| Quality of life | 96.21 | 21.02 | -0.713* | -0.657** | 0.399** | 1 | -0.665** |
| Depression | 7.48 | 4.94 | 0.682** | 0.673** | -0.352** | -0.665** | 1 |

**Note**: **p<0.01, *p<0.05. M, mean; SD, standard deviation.

Supplementary Table 2: Robustness analysis by using the moderator variable social support instead of marital relations (N=802).

**Table 2a. Regression coefficients for moderated mediation models**

|  |  | Model 1 | Model 2 | Model 3 | Model 4 |
| --- | --- | --- | --- | --- | --- |
| Control variables | Cervical cancer stage | 0.0093  (-0.1768~0.2219) | 0.0672  (-0.1539~0.3185) | 0.0915  (-0.2059~0.3649) | 0.0438  (-0.3091~0.2143) |
|  | Chemotherapy course | 0.0225  (-0.0426~0.2137) | 0.1086*  (0.0121~0.2987) | 0.1428*  (0.0723~0.3233) | 0.2011*  (0.0879~0.4237) |
|  | Number of radiation treatments | -0.0092  (-0.02759~0.0125) | -0.0210  (-0.0441~0.0115) | -0.0333  (-0.0524~0.0109) | -0.0306*  (-0.0519~-0.0073) |
| Independent variables | Digestive system-related symptom cluster | 0.0236*  (0.0131~0.1508) |  |  |  |
|  | Dizziness-ringing in the ears |  | 0.331*  (0.2081~0.4989) |  |  |
|  | Skin dryness and itching |  |  | 0.5692*  (0.2413~0.8769) |  |
|  | Urinary frequency-urgency-leakage |  |  |  | 0.4101*  (0.0103~0.7121) |
| Mediating variables | Neuroticism | 0.3382**  (0.1501~0.5327) | 0.2561**  (0.067~0.4451) | 0.4241**  (0.2149~0.6059) | 0.2801*  (0.1003~0.4781) |
| Moderating variables | Quality of life | -0.2861  (-0.0771~0.0141) | -0.0619**  (-0.0593~-0.0207) | -0.0188  (-0.0805~0.0216) | -0.0479  (-0.131~0.0232) |
|  | Social support | -0.0235  (-0.0536~0.0175) | -0.0265  (-0.0618~0.0169) | -0.0538*  (-0.101~-0.0214) | -0.0445  (-0.094~0.0117) |
| Interactions | Adverse reactions*social support | 0.0110  (-0.0093~0.0102) | -0.0081  (-0.0134~0.0129) | 0.0075  (-0.0042~0.013) | 0.0011  (-0.0023~0.0071) |
|  | Neuroticism*social support | 0.0013  (-0.0097~0.0105) | 0.0190*  (0.0036~0.0304) | 0.0015*  (0.0009~0.0047) | 0.0012*  (0.0007~0.0111) |
|  | Adverse reactions*quality of life | 0.0028  (-0.0004~0.0107) | 0.0221**  (0.0104~0.0372) | 0.0205**  (0.0123~0.0452) | 0.0038  (-0.0009~0.0125) |
|  | Neuroticism*quality of life | -0.0011  (-0.0026~0.0052) | -0.0031  (-0.0053~0.0021) | -0.0048**  (-0.0077~-0.0101) | -0.0024  (-0.0051~0.0023) |
| R2 | | 0.611 | 0.604 | 0.587 | 0.569 |
| Sig | | <0.001 | <0.001 | <0.001 | <0.001 |
| F value | | 119.233 | 92.321 | 79.012 | 72.908 |
| Dependent variable: Depression | | | | | |

**Note:** **P<0.01, *P<0.05.

**Table 2b. Indirect effects moderated by marital relations and quality of life**

|  | Effect type | Social support | | | |
| --- | --- | --- | --- | --- | --- |
|  |  | Low | Low | High | High |
|  |  | Quality of life | | | |
|  |  | Low | High | Low | High |
| Model 1 | Indirect | 0.0398  (-0.011~0.215) | 0.0102  (-0.0296~0.163) | 0.0614  (-0.021~0.232) | 0.0123  (-0.051~0.194) |
|  | Direct | 0.3715**  (0.176~0.5762) | 0.502**  (0.263~0.723) | 0.4002**  (0.193~0.817) | 0.4932**  (0.298~0.859) |
|  | Total | 0.4113**  (0.163~0.693) | 0.5122**  (0.265~0.873) | 0.4616**  (0.182~0.773) | 0.5055**  (0.271~0.892) |
| Model 2 | Indirect | 0.0086  (-0.014~0.223) | -0.0617  (-0.159~0.175) | 0.1213  (-0.039~0.367) | 0.0057  (-0.183~0.206) |
|  | Direct | 0.5732**  (0.311~0.853) | 1.065**  (0.801~1.511) | 0.4575*  (0.115~0.832) | 0.926**  (0.606~1.473) |
|  | Total | 0.5818**  (0.241~0.821) | 1.0033**  (0.622~1.531) | 0.5788*  (0.051~0.982) | 0.9317**  (0.891~1.441) |
| Model 3 | Indirect | 0.0197  (-0.108~0.258) | 0.0254  (-0.078~0.151) | 0.321  (-0.167~0.451) | 0.0173  (-0.089~0.282) |
|  | Direct | 0.1295  (-0.287~0.383) | 0.487**  (0.181~0.786) | 0.0616  (-0.295~0.273) | 0.4167**  (0.156~0.851) |
|  | Total | 0.1492  (-0.324~0.341) | 0.5124**  (0.187~0.856) | 0.3826  (-0.482~0.413) | 0.434**  (0.242~0.832) |
| Model 4 | Indirect | 0.1051*  (0.035~0.186) | 0.0631  (-0.089~0.314) | 0.2369*  (0.132~0.359) | 0.1361*  (0.045~0.249) |
|  | Direct | 0.0712  (-0.038~0.4215) | 0.2531**  (0.016~0.523) | 0.2087  (-0.013~0.463) | 0.3001**  (0.125~0.506) |
|  | Total | 0.1763*  (0.026~0.427) | 0.3162**  (0.049~0.437) | 0.4456*  (0.082~0.691) | 0.4362**  (0.145~0.749) |

**Note: **P<0.01, *P<0.05.**  In Models 1–4, the PHQ-9 scale score is a dependent variable, the NEO-FFIN scale score is a mediating variable, and the FACT-Cx and Perceived Social Support Scale scores are moderating variables. In Model 1, the digestive system-related symptom cluster is the independent variable; in Model 2, dizziness-ringing in the ears is the

independent variable; in Model 3, skin dryness and itching is the independent variable; and in Model 4, urinary frequency-urgency-leakage is the independent variable.

Supplementary Table 3: Robustness analysis by using maximum likelihood estimation instead of least squares estimation (N=802).

**Table3a . Regression coefficients for moderated mediation models**

|  |  | Model 1 | Model 2 | Model 3 | Model 4 |
| --- | --- | --- | --- | --- | --- |
| Control variables | Cervical cancer stage | 0.0183  (-0.2932~0.3336) | 0.0569  (-0.2747~0.3903) | 0.0839  (-0.2452~0.4146) | 0.0523  (-0.2972~0.4031) |
|  | Chemotherapy course | 0.0376  (-0.0609~0.1351) | 0.1062  (0.002~0.2069) | 0.1049*  (0.0032~0.2067) | 0.1642*  (0.0572~0.2695) |
|  | Number of radiation treatments | -0.021  (-0.0347~0.0006) | -0.0196  (-0.037~-0.0011) | -0.022  (-0.0378~0.0007) | -0.0281  (-0.0472~-0.0076) |
| Independent variables | Digestive system-related symptom cluster | 0.0231*  (0.0101~0.1492) |  |  |  |
|  | Dizziness-ringing in the ears |  | 0.352*  (0.1959~0.5025) |  |  |
|  | Skin dryness and itching |  |  | 0.5722*  (0.2379~0.9047) |  |
|  | Urinary frequency-urgency-leakage |  |  |  | 0.4001*  (0.0992~0.6995) |
| Mediating variables | Neuroticism | 0.3325**  (0.1523~0.5121) | 0.2423**  (0.0582~0.4249) | 0.4142**  (0.2339~0.5952) | 0.272*  (0.0882~0.4611) |
| Moderating variables | Quality of life | -0.0313  (-0.0852~0.0242) | -0.0761**  (-0.1359~-0.0167) | -0.0189  (-0.0769~0.0392) | -0.061  (-0.109~0.0123) |
|  | Marital relations | -0.0289  (-0.0779~0.0212) | -0.0352  (-0.0886~0.0223) | -0.0582*  (-0.1112~-0.0042) | -0.0523  (-0.1067~0.013) |
| Interactions | Adverse reactions*marital relations | 0.0012  (-0.0009~0.0021) | -0.0042  (-0.0121~0.0062) | 0.0021  (-0.0043~0.012) | 0.0019  (-0.0028~0.0059) |
|  | Neuroticism*marital relations | 0.0007  (-0.002~0.005) | 0.003  (-0.0004~0.0031) | 0.0023*  (0.0003~0.011) | 0.0017  (-0.0006~0.0031) |
|  | Adverse reactions*quality of life | 0.0042  (-0.0011~0.0081) | 0.0152**  (0.0052~0.0264) | 0.0181**  (0.0102~0.0256) | 0.0052  (-0.0022~0.0126) |
|  | Neuroticism*quality of life | -0.0035  (-0.011~0.0009) | -0.0012  (-0.0028~0.0023) | -0.0041**  (-0.0051~-0.0024) | -0.0026  (-0.0041~0.0027) |
| Chi-square/df | | 2.642 | 1.983 | 2.873 | 2.763 |
| CFI | | 0.909 | 0.912 | 0.901 | 0.900 |
| AGFI | | 0.913 | 0.916 | 0.902 | 0.905 |
| RMSEA | | 0.064 | 0.056 | 0.072 | 0.068 |
| Dependent variable: Depression | | | | | |

**Note:** Chi-square/df < 3, CFI > 0.9, AGFI > 0.9 and RMSEA < 0.08, which indicates the model fits well.

**Table 3b. Indirect effects moderated by marital relations and quality of life**

|  |  | Marital relations | | | |
| --- | --- | --- | --- | --- | --- |
|  | Effect type | Low | Low | High | High |
|  |  | Quality of life | | | |
|  |  | Low | High | Low | High |
| Model 1 | Indirect | 0.0449  (-0.013~0.126) | 0.0034  (-0.053~0.071) | 0.0582  (-0.023~0.145) | 0.0087  (-0.039~0.071) |
|  | Direct | 0.3623**  (0.249~0.462) | 0.482**  (0.329~0.613) | 0.3967**  (0.249~0.513) | 0.5069**  (0.411~0.632) |
|  | Total | 0.4072**  (0.243~0.583) | 0.4854**  (0.289~0.671) | 0.4549**  (0.262~0.653) | 0.5156**  (0.353~0.669) |
| Model 2 | Indirect | 0.0011  (-0.007~0.113) | -0.081  (-0.239~0.079) | 0.1022  (-0.047~0.281) | 0.0043  (-0.159~0.168) |
|  | Direct | 0.5721**  (0.313~0.826) | 1.062**  (0.807~1.461) | 0.4559*  (0.101~0.812) | 0.952**  (0.807~1.272) |
|  | Total | 0.5732**  (0.229~0.823) | 0.981**  (0.832~1.538) | 0.5581*  (0.039~0.872) | 0.9563**  (0.889~1.441) |
| Model 3 | Indirect | 0.0286  (-0.123~0.061) | 0.022  (-0.086~0.082) | 0.0267  (-0.126~0.078) | 0.0173  (-0.121~0.091) |
|  | Direct | 0.1023  (-0.253~0.076) | 0.479**  (0.176~0.791) | 0.092  (-0.287~0.142) | 0.4953**  (0.237~0.739) |
|  | Total | 0.1309  (-0.384~0.162) | 0.501**  (0.175~0.869) | 0.1187  (-0.424~0.236) | 0.5126**  (0.232~0.846) |
| Model 4 | Indirect | 0.1039*  (0.032~0.187) | 0.0362  (-0.039~0.142) | 0.2369*  (0.125~0.343) | 0.1374*  (0.039~0.252) |
|  | Direct | 0.0883  (-0.047~0.258) | 0.2539**  (0.011~0.525) | 0.158  (-0.061~0.342) | 0.2979**  (0.111~0.478) |
|  | Total | 0.1922*  (0.019~0.434) | 0.2901**  (0.039~0.458) | 0.3949*  (0.076~0.692) | 0.4353**  (0.136~0.757) |

**Note: **P<0.01, *P<0.05.**  In Models 1–4, the PHQ-9 scale score is a dependent variable, the NEO-FFIN scale score is a mediating variable, and the FACT-Cx and LWSMAT scores are moderating variables. In Model 1, the digestive system-related symptom cluster is the independent variable; in Model 2, dizziness-ringing in the ears is the independent variable; in Model 3, skin dryness and itching is the independent variable; and in Model 4, urinary frequency-urgency-leakage is the independent variable.
